# MsGf: A Lightweight Self-Supervised Monocular Depth Estimation Framework with Multi-Scale Feature Extraction

**DOI:** 10.3390/s25206380

**Published:** 2025-10-16

**Authors:** Xinxing Tian, Zhilin He, Yawei Zhang, Fengkai Liu, Tianhao Gu

**Affiliations:** School of Automation, Qingdao University, Qingdao 266071, China; 2778707683@qdu.edu.cn (X.T.); hezhilin@qdu.edu.cn (Z.H.); zhangyawei@qdu.edu.cn (Y.Z.); liufengkai1@qdu.edu.cn (F.L.)

**Keywords:** edge feature extraction, guided filtering, lightweight model, multi-scale feature extraction, self-supervised monocular depth estimation

## Abstract

Monocular depth estimation is an essential component in computer vision that enables 3D scene understanding, with critical applications in autonomous driving and augmented reality. This paper proposes a lightweight self-supervised framework from single RGB images for multi-scale feature extraction and artifact elimination in monocular depth estimation (MsGf). The proposed framework first designs a Cross-Dimensional Multi-scale Feature Extraction (CDMs) module. The CDMs module combines parallel multi-scale convolution with sequential feature convolutions to achieve multi-scale feature extraction with minimal parameters. Additionally, a Sobel Edge Perception-Guided Filtering (SEGF) module is proposed. The SEGF module uses the Sobel operator to decompose the features into horizontal direction features and vertical direction features, and then generates the filter kernel through two steps of filtering to effectively suppress artifacts and better capture structural and edge features. A large number of ablation experiments and comparative experiments on the KITTI and Make3D datasets demonstrate that the MsGf with only 0.8 M parameters can achieve better performance than the current most advanced methods.

## 1. Introduction

Depth estimation, which aims to recover 3D geometric scene information from 2D images or videos, is a critical technology for constructing visual perception systems. Its performance directly impacts environmental understanding and decision-making in applications such as autonomous driving, augmented reality, and mobile robotics. The research on image depth estimation has shifted from traditional methods that relied on geometric principles [1,2,3] to a technology route dominated by deep learning, which has achieved superior performance thanks to its powerful representation learning capabilities [4]. Particularly in edge computing scenarios—such as autonomous vehicles requiring low latency and high energy efficiency, and augmented reality devices demanding real-time response and portability—depth estimation models must not only ensure accuracy but also strictly comply with real-time operation, low power consumption, and stringent constraints on computational resources.

The early depth estimation methods relied mainly on supervised learning, which can achieve millimeter-level precision using LiDAR sensors [5]. However, their high deployment costs and strong dependence on annotated data significantly limit the broad applicability of supervised approaches. Consequently, unsupervised learning methods have emerged as a promising alternative, primarily encompassing stereo and monocular depth estimation [6,7,8]. Although stereo depth estimation has potential for higher accuracy [9], it requires synchronized operation of dual cameras, involves complex calibration procedures, and suffers from computationally expensive disparity matching, often hindering real-time performance. In contrast, self-supervised monocular depth estimation utilizes only a single camera. Its advantages of low hardware cost and high computational efficiency have made it an increasingly active research area [10].

Prevailing self-supervised monocular depth estimation models are characterized by their substantial computational burden and high parameter count, leading to a significant disparity between the model’s requirements and the limited resources available on edge hardware [11]. Previous studies have attempted to reduce the computational complexity of the model by lightweight designs such as reducing the number of network layers or channels [12], which can lead to a decline in the model’s ability to extract multi-scale features. But the performance of monocular depth estimation largely depends on the network’s ability to extract effective features. Multi-scale features are crucial for understanding objects and structures of different sizes in the scene. Due to the limitation of local receptive fields, traditional convolutional neural networks have difficulty effectively capturing large-scale scene context information, resulting in significant deviations in depth prediction in weak texture regions such as smooth walls and the sky [13]. Conversely, while transformer-based methods excel at modeling global dependencies, they frequently neglect fine-grained local geometric details [14], resulting in compromised precision for small structures and object boundaries.

Additionally, artifacts are a key factor restricting monocular depth estimation [15]. They arise partly from reduced model parameters, which prevent accurate depth information restoration [16]. Also, current spatial geometric constraints and feature representation methods are primarily optimized for capturing larger-scale structures [17]. For small objects and intricate details, the inherent limitations in multi-scale feature extraction make their edges unclear in depth estimation. This insufficient representation of fine-grained features not only degrades the accuracy for small regions but also exacerbates the generation of artifacts along object boundaries and in areas with complex textures.

**Figure 1:**
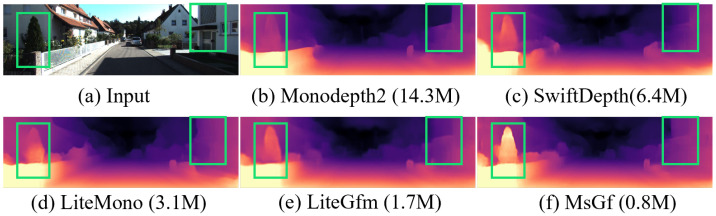
The proposed MsGf has fewer parameters than others but generates more accurate depth maps.

To address these issues, this paper focuses on lightweight self-supervised monocular depth estimation, aiming to overcome the limitations of current models in feature extraction and artifact reduction. We propose a novel self-supervised depth estimation framework that combines lightweight multi-scale feature extraction with artifact elimination. The framework consists of two key modules. First, the Cross-Dimensional Multi-scale Feature Extraction (CDMs) module addresses the limitations of feature extraction capability and model complexity by effectively compressing parameters using parallel multi-scale convolutional kernel groups while accurately capturing multi-scale image features. Second, the Sobel Edge Perception-Guided Filtering (SEGF) module specifically targets the problem of artifacts by leveraging Sobel operators to generate the horizontal direction features ($F_{hor}$) and the vertical direction features ($F_{ver}$), subsequently producing filtering kernels through two-step filtering operations. As shown in Figure 1, this approach suppresses artifacts while efficiently capturing structural and edge features.

In summary, the main contributions of this paper are as follows.

1. The paper designs the MsGf framework. It drastically reduces the number of parameters while retaining key details, effectively suppressing artifacts and enhancing feature extraction. With only 0.8 M trainable parameters, it achieves optimal performance in the KITTI and Make3D datasets [18].
2. The paper proposes a CDMs module. It uses multi-scale feature stitching and fusion strategies to reduce parameters and expand the receptive field. This approach diminishes detail loss, suppresses redundant information during multi-scale fusion, and inhibits edge artifacts.
3. The paper introduces an SEGF module. This module extracts features using a Sobel operator and filters RGB images in the encoder. By cross-modal fusion of depth and RGB features, it effectively addresses structural loss during image processing.

## 2. Related Works

### 2.1. Self-Supervised Monocular Depth Estimation

Due to the high difficulty in obtaining large-scale real-depth values, self-supervised monocular depth estimation methods attract significant attention from researchers. Godard et al. [19] model the depth estimation problem as a view synthesis problem and train the depth network model by minimizing the photometric loss between the original image and the reconstructed image. Islam et al. [20] proposed a monocular depth estimation method based on generative adversarial networks, which improves the accuracy of depth estimation by using a pre-trained GAN model. The USegDepth model achieves cross-task feature interaction through the shared encoder of semantic segmentation and depth estimation [21], but the dual-task design results in a large number of parameters. The S2R-Depth model [22] proposes a structure-style decoupling module and depth-specific attention, improving the cross-domain generalization ability, but artifacts still exist in depth discontinuous areas. Chen et al. [23] propose a joint self-supervised learning framework, improving the accuracy of depth prediction in challenging scenarios through the collaborative optimization of depth and optical flow estimation, but depth estimation errors still occur for small objects and in severely occluded regions. With the deepening of research, multi-task collaborative frameworks and loss function optimization methods develop in parallel [24,25,26]. The SC-Depth series of models [27,28,29] introduces pre-trained networks to generate depth priors and subsequently strengthens the processing ability for complex scene elements through optimizing the object masker, but depth prediction at object boundaries remains blurry and prone to artifacts. The Monodepth series of works [19] addresses occlusion and interference issues in complex scenes through minimizing the re-projection loss and the automatic mask mechanism, yet struggles to eliminate boundary artifacts and detail loss.

The above models all prioritize accuracy and scene robustness as their primary goals, with large parameter quantities at the expense of computational efficiency. They are difficult to deploy effectively on resource-constrained edge-end devices. The Lite-Mono model [30] expands the receptive field by continuously expanding convolution to replace deepening the network and designs a local–global feature interaction module to achieve global modeling with a lightweight attention mechanism. Yang et al. [31] improved the accuracy and details of depth estimation by integrating depth maps of different scales, effectively capturing multi-scale information in the scene and enhancing the performance of depth estimation. The MobileDepth [32] model introduces a feature distillation framework guided by motion cues to enhance the structural consistency of Lite-Mono [30] in scenes with complex spatial relationships, particularly improving depth coherence for objects exhibiting displacement artifacts. It guides the MobileNetV3 [33] model to learn motion features using the optical flow prediction of the ManyDepth [34] model without increasing the parameter quantity. The RA-Depth [35] model integrates a resolution adaptive mechanism and dual lightweight HRNet to alleviate the problem of model performance decline in different lighting conditions of MobileDepth [32]. MG-Mono [36] achieves efficient extraction of pixel-level, local, and global features through its Multi-Granularity Information Fusion (MGIF) module. RTIA-Mono [37] introduces a real-time lightweight architecture that aggregates global and local information via an attention-guided mechanism.

These methods further strike a balance between parameter compression and feature representation capability. However, they still exhibit edge blurring and depth discontinuities in weakly textured regions, and it remains highly challenging to simultaneously preserve fine details and suppress artifacts under extremely low parameter budgets. Therefore, this paper proposes a lightweight multi-scale feature extraction module, which accurately extracts feature information through a local–global feature coupling mechanism while maintaining a low parameter level.

### 2.2. Guided Filter

The development of guided filtering begins with the innovation of traditional bilateral filtering [38]. He and others build a basic framework with a local linear model [39], which reduces the complexity of the computation and avoids gradient reversal artifacts. However, the method still suffers from edge blurring due to its assumption of uniform areas. To solve this, edge-aware weighting [40] and gradient domain optimization [41] are used. They enhance structure preservation with edge constraints, but high-frequency detail loss remains in complex textured scenes. Superpixel segmentation [42] then emerges, improving local consistency, but its fixed segmentation mechanism restricts multi-scale adaptability. In recent years, deep learning-based methods such as dual-branch networks [43] and multi-scale dynamic kernels [44] have emerged. They strengthen feature expression through data-driven strategies. However, the complex structure of the network leads to increased computation costs. GroupViT [45] makes a breakthrough in semantic understanding but has high memory demands due to the Transformer architecture. Despite attempts to balance efficiency and accuracy with lightweight designs [46,47,48,49], existing methods often face high-frequency information degradation due to model compression.

In this paper, depth prediction is guided by the structural features of RGB images, effectively suppressing edge artifacts in depth maps.

## 3. Methods

### 3.1. MSGF Framework

**Figure 2:**
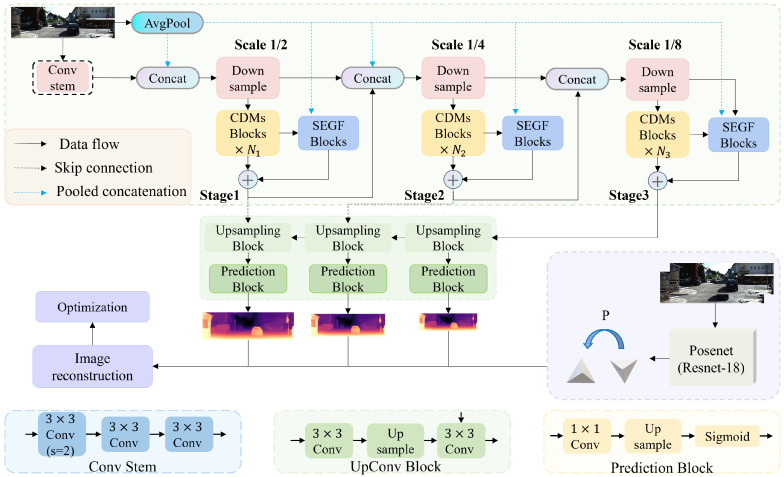
Regarding the overview of the proposed MsGf. MsGf consists of an encoder–decoder deep network for deep prediction, as well as a commonly used PoseNet for estimating the pose between adjacent monocular frames. The encoder of the deep network is composed of four stages. Features of three scales are fused through upsampling and channel concatenation, and rich hierarchical features are extracted using CDMs modules and SEGF modules. The detailed information of these modules is shown in Figure 3.

This paper presents a lightweight self-supervised monocular depth estimation framework, MsGf. As shown in Figure 2, the framework comprises a depth network (DepthNet) and a pose network (PoseNet). To address the issues of artifacts and edge blurring in the depth maps produced by existing lightweight networks, we design a lightweight CDMs module and an SEGF module in the DepthNet encoder. These modules enhance structural information and preserve edge details through multi-scale feature fusion and a dynamic filtering mechanism (see Section 3.3 for details).

DepthNet. We present an improved lightweight encoder–decoder architecture for DepthNet, with CDMs and SEGF modules at its core. The CDMs module uses a multi-branch parallel structure and depth-wise separable convolutions to achieve a lightweight design and efficient multi-scale feature fusion. The SEGF module dynamically generates spatially adaptive filtering kernels. It filters by combining edge information from the guide image, effectively alleviating the edge detail loss and cross-modal data mismatch common in traditional methods.

PoseNet. PoseNet takes RGB image pairs as input. It uses a pre-trained ResNet18 as the encoder to extract image features [19,29], and a four-layer convolutional pose decoder to estimate the camera’s 6-DoF pose parameters and depth maps.

### 3.2. Cross-Dimensional Multi-Scale Feature Extraction Module

**Figure 3:**
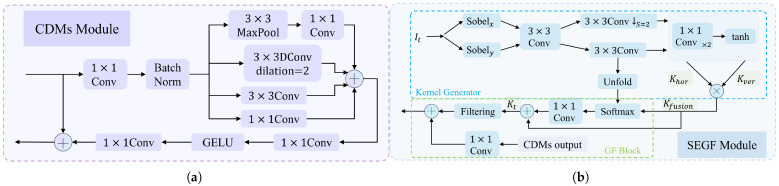
Figure (**a**) shows the structure of the CDMs module, and figure (**b**) shows the structure of the SEGF module. In each stage, the CDMs module with a different dilation rate is repeated N times.

Traditional CNNs, which employ small convolutional kernels, exhibit a limited receptive field and struggle to capture the extensive contextual information required for inferring depths in areas such as skies or smooth walls [29]. In contrast, the CDMs module enhances the perception of multi-scale context by utilizing parallel multi-scale convolutions and dilated convolutions. This design significantly expands the network’s receptive field, thereby improving its ability to interpret weakly textured regions. As shown in Figure 3, to reduce the number of parameters, this paper replaces the consecutive expansion convolution with a 1×1 convolution. Subsequently, a parallel multi-scale convolution kernel group is introduced, and the 5×5 convolution is replaced with a dilated convolution with a dilation rate of 2:

(1) $$F_{3\times 3}^{\mathrm{DC}}={\mathrm{DConv}}_{3\times 3}\left(\mathrm{dilation}=2\right)$$

Different from the isolated computation of traditional parallel structures, collaborative optimization is achieved through cross-scale weight sharing of feature maps. A local–global feature collaborative perception mechanism is established. After the outputs of each branch are concatenated along the channel dimension, the channels are compressed through two 1×1 convolutions, and multi-scale information is fused:

(2) $$F_{\mathrm{fuse}}={\mathrm{Conv}}_{1\times 1}\left(\mathrm{GELU}\left({\mathrm{Conv}}_{1\times 1}\left(\mathrm{Concat}\left(F_{1\times 1},\;F_{3\times 3},\;F_{3\times 3}^{\mathrm{DC}},\;F_{\mathrm{maxpool}}\right)\right)\right)\right)$$

Batch normalization and GELU activation functions are incorporated. They improve training stability, reduce parameters, and improve the representation of details and structural information.

### 3.3. Dynamic Guided Filtering Framework

#### 3.3.1. Sobel Edge Perception-Guided Filtering Module

To address edge blur and artifacts in lightweight models, a guided filtering module is introduced to enhance image details and suppress noise. The output of the downsample layer in each stage serves as the target feature $I_{t}\in R^{H\times W\times C^{t}}$, while the merged image with the full features acts as the guide feature $I_{g}\in R^{H\times W\times C^{g}}$, where *H*, *W*, and *C* denote height, width, and channels, respectively. The filtering kernel $K_{t}\in R^{H\times W\times k\times k}$ is generated from $I_{t}$ and $I_{g}$. Using prior information from the guided kernel, the input features are reconstructed to retain edges and gradients.

The entire guided process of this filtering kernel is expressed as follows:

(3) $$\begin{array}{cc} I_{3} & =\;\mathrm{Conv}\left(\mathrm{Concat}\left(I_{g}^{3},\;F_{2}\right)\right),\;F_{3}=\mathrm{SEGF}\left[I_{3},\;K_{t3}\right]\\ I_{2} & =\;\mathrm{Conv}\left(\mathrm{Concat}\left(I_{g}^{2},\;F_{1}\right)\right),\;F_{2}=\mathrm{SEGF}\left[I_{2},\;K_{t2}\right]\\ I_{1} & =\mathrm{Conv}\;\left(\mathrm{Concat}\left(I_{g}^{1},\;I_{c}\right)\right),\;F_{1}=\mathrm{SEGF}\left[I_{1},\;K_{t1}\right] \end{array}$$

where $I_{i}$ and $K_{ti}$ represent the input of the *i*-th filtering process in the SEGF module, SEGF[·, ·] is the guided filter for the corresponding features. For the feature *i*, the filtering process is as follows:

(4) $$F_{filtered}^{i}=einsum\left(hwkc,\;hwc\to hwk,\;K_{t}i,\;I_{i}\right)$$

The CDMs output feature and the filtering results are fused via residual connections.

(5) $$F_{i}=F_{\mathrm{filtered}}^{i}+{\mathrm{Conv}}_{1\times 1}\left(F_{\mathrm{CDMs}}\right)$$

Filtered features $F_{i}$, 0≤i<m, are designed to capture more details and are fed into the network to generate higher-quality depth maps. Next, we detail the guided image kernel generator and the loss function designed for network training.

#### 3.3.2. Guided Filter Kernel Generator

As shown in Figure 3, the target feature and the guided feature are used as input. Due to the fact that the depth discontinuity mainly manifests as the directional edge response in the image space, in order to accurately capture this geometric characteristic, we have designed a direction-enhanced Sobel operator [50]. Its horizontal and vertical kernels are set to [[3, 0, −3], [10, 0, −10], [3, 0, −3]] and [[3, 10, 3], [0, 0, 0], [−3, −10, −3]], respectively. Then, pooling operations are performed to obtain $F_{x}^{t}$ and $F_{y}^{t}$. This process is expressed as:

(6) $$\begin{array}{c} F_{x}^{t}=\mathrm{Avg}\left({\mathrm{Sobel}}_{x}\left(\mathrm{Conv}(I_{t})\right)\right)\\ F_{y}^{t}=\mathrm{Avg}\left({\mathrm{Sobel}}_{y}\left(\mathrm{Conv}(I_{t})\right)\right) \end{array}$$

By using adaptive learning convolution to align the RGB edges and the discontinuous depth areas, the edge features in two directions are concatenated and nonlinearly fused:

(7) $$F_{\mathrm{fused}}=\sigma \left(C_{3\times 3}\left(\left[F_{x}^{t};F_{y}^{t}\right]\right)\right)$$

where [·; ·] denotes channel-wise concatenation, $C_{3\times 3}$ is a 3 × 3 convolutional layer, and σ is the PReLU activation function. The fused feature $F_{fused}$ generates the horizontal direction features $F_{hor}$ and the vertical direction features $F_{ver}$ through a two-branch structure.

(8) $$\begin{array}{cc} F_{\mathrm{hor}} & =C_{3\times 3}^{↓2}\left(F_{\mathrm{fused}}\right)\\ F_{\mathrm{ver}} & =C_{3\times 3}\left(F_{\mathrm{fused}}\right) \end{array}$$

where $C_{3\times 3}^{↓2}$ represents a 3 × 3 downsampling convolution with a stride of 2, and $C_{3\times 3}$ is a convolution with a stride of 1. Both include PReLU activation.

Two layers of 1 × 1 convolutions and a tanh function are applied to $F_{\mathrm{hor}}$ and $F_{\mathrm{ver}}$ to generate the preliminary kernel:

(9) $$\begin{array}{cc} K_{\mathrm{hor}} & =\tanh\left(C_{1\times 1}\left(C_{1\times 1}\left(F_{\mathrm{hor}}\right)\right)\right)\\ K_{\mathrm{ver}} & =\tanh\left(C_{1\times 1}\left(C_{1\times 1}\left(F_{\mathrm{ver}}\right)\right)\right)\\ K_{\mathrm{fusion}} & =\frac{K_{\mathrm{hor}}⊙K_{\mathrm{ver}}}{\alpha \cdot \sqrt{K^{2}+\epsilon }} \end{array}$$

where $C_{1\times 1}$ is a 1 × 1 convolutional layer with $k^{2}$ output channels; ⊙ denotes element-wise multiplication, which achieves collaborative enhancement of horizontal and vertical edges; α is a learnable scale factor, dynamically adjusting the intensity of detail retention; $\epsilon =1\times 10^{-8}$ is a small value; the function tanh constrains the kernel weights to [−1, 1] to prevent numerical instability during filtering.

Next, high-resolution features enhance the kernel’s spatial adaptability:

(10) $$U_{\mathrm{ver}}=\mathrm{Unfold}\left(F_{\mathrm{ver}},k\right)\in R^{C_{g}\times k^{2}\times H\times W}$$

(11) $$K_{r}=\mathrm{Softmax}\left(\sum_{c=1}^{C_{g}}K_{\mathrm{fusion}}\cdot U_{\mathrm{ver}}\left(c\right)\right)$$

where $C_{1\times 1}$ is a 1 × 1 convolutional layer with $k^{2}$ output channels; ⊙ denotes element-wise multiplication, which achieves collaborative enhancement of horizontal and vertical edges; α is a learnable scale factor, dynamically adjusting the intensity of detail retention; $\epsilon =1\times 10^{-8}$ is a small value; the function tanh constrains the kernel weights to [−1, 1] to prevent numerical instability during filtering. Next, high-resolution features enhance the kernel’s spatial adaptability: $K_{t}$:

(12) $$K_{t}=K_{\mathrm{fusion}}+C_{1\times 1}\left(K_{r}\right)$$

### 3.4. Self-Supervised Learning

This paper uses a self-supervised learning framework for monocular depth estimation, employing geometric consistency constraints between temporal frames to facilitate joint optimization of depth and pose. The core process is as follows.

An RGB target image $I_{t}\in R^{H\times W\times 3}$ is input, and DepthNet outputs an inverse depth map per pixel. Meanwhile, PoseNet estimates the relative pose of the camera $T_{t\to s}$ from the adjacent frame {I (t − 1), I (t + 1)}. Through projection transformation, the source image is assigned to the target view according to depth and pose, generating a reconstructed image ${\hat{I}}_{s\to t}$. The projection formula is defined as:

(13) $${\hat{I}}_{s\to t}=I_{s}\langle \mathrm{proj}\left(D_{t},\;T_{t\to s},\;K\right)\rangle$$

where *K* is the intrinsic matrix of the camera, and 〈·〉 denotes the bilinear sampling operation.

Image Reconstruction Loss. The reprojection loss between an image and its previous or next frame can be expressed as:

(14) $$L_{p}\left(I_{s},\;I_{t}\right)=\min_{I_{s}\in \left[-1,1\right]}L_{p}\left(I_{s\to t},\;I_{t}\right)$$

where $L_{p}$ is the photometric loss, combining SSIM [51] and $L_{1}$ to measure the difference between the reconstructed ${\hat{I}}_{s\to t}$ and target images $I_{t}$:

(15) $$L_{p}\left(I_{s\to t},\;I_{t}\right)=\alpha \frac{1-\mathrm{SSIM}({\hat{I}}_{s\to t},\;I_{t})}{2}+\left(1-\alpha \right){∥{\hat{I}}_{s\to t}-I_{t}∥}_{1}$$

where α=0.85 balances the two terms.

By minimizing multi-frame reconstruction errors, occluded areas and dynamic objects are adaptively removed:

(16) $$\mu =\min_{I_{s}\in \left[-1,1\right]}L_{p}\left(I_{s},\;I_{t}\right)>\min_{I_{s}\in \left[-1,1\right]}L_{p}\left({\hat{I}}_{s\to t},\;I_{t}\right)$$

Thus, the image reconstruction loss is:

(17) $$L_{r}\left({\hat{I}}_{s\to t},\;I_{t}\right)=\mu \cdot L_{p}\left(I_{s},\;I_{t}\right)$$

Edge-Aware Smoothness Loss. This loss constrains local depth map smoothness while preserving object boundaries:

(18) $$L_{\mathrm{smooth}}=|\partial_{x}d_{t}^{*}|e^{-|\partial_{x}I_{t}|}+\left|\partial_{y}d_{t}^{*}\right|e^{-|\partial_{y}I_{t}|}$$

where $d_{t}^{*}=\frac{d_{t}}{{\bar{d}}_{t}}$ is the normalized inverse depth, and ${\bar{d}}_{t}$ is the mean depth.

Boundary-Aware Loss. Since the aforementioned per-pixel optimization treats all pixels equally, it cannot effectively retain high-frequency structural details and causes detail artifacts in depth maps. To solve this and encourage the network to prioritize high-frequency components, a boundary-aware loss is introduced, enhancing the model’s ability to generate clearer boundaries and complex details. As in [52], it is defined as follows:

(19) $$L_{ba}={∥M⊙(I_{t}-{\hat{I}}_{s\to t})∥}_{1}$$

where ⊙ denotes element-wise multiplication, and the boundary mask *M* is generated by extracting edge differences between the target frame and reconstructed image using the Sobel operator:

(20) $$M_{s}=\left(\nabla_{x}{\hat{I}}_{s}-\nabla_{x}I_{s}\right)⊙\left(\nabla_{y}{\hat{I}}_{s}-\nabla_{y}I_{s}\right)$$

where $\nabla_{x},\nabla_{y}$ are horizontal and vertical Sobel kernels, ⊙ represents element-wise multiplication.

The total loss function, combining the three losses, is

(21) $$L=\lambda_{1}L_{p}+\lambda_{2}L_{\mathrm{smooth}}+\lambda_{3}L_{ba}$$

where $\lambda_{1},\lambda_{2},\lambda_{3}$ are set to 1, $10^{-3}$, and 0.02, respectively.

## 4. Results

This section first details the implementation of the network architecture and training strategy and then conducts quantitative performance verification using the KITTI, Make3D, and NYU Depth V2 datasets. To fully explore the effectiveness of the model design, ablation studies are performed to evaluate each innovative module’s contribution. Finally, multi-scenario visual comparisons reveal each component’s differential impact on depth prediction accuracy and edge consistency.

### 4.1. Implementation Details

#### 4.1.1. Dataset

This paper uses the KITTI dataset [18] as the core benchmark and follows the Eigen split protocol [11], dividing the data into 39,810 training, 4424 validation, and 697 test images, aligning with methods such as Monodepth2. Input images are scaled to 192 × 640, with a depth prediction range of 0–80 m for autonomous driving scenarios. Furthermore, to test the generalization performance, the Make3D dataset [53], which consists of 134 test images of outdoor scenes, is also used to evaluate the performance of the models trained on the KITTI dataset. Here, following common practices, the depth prediction range is defined as 0 to 80 m.

#### 4.1.2. Hyperparameters

The model is built in the PyTorch framework (version 1.10.1) and trained on an NVIDIA 3090 GPU with a batch size of 12. The AdamW optimizer conducts gradient descent with a weight decay coefficient of 0.01. All experiments are trained from scratch for 60 epochs. The initial learning rate is 0.0005, switched to 0.0001 for fine-tuning from epoch 31, taking about 18 h.

#### 4.1.3. Evaluation Metrics

Seven evaluation metrics from the literature [52] are used: Absolute Relative Error (Abs Rel), Squared Relative Error (Sq Rel), Root Mean Squared Error (RMSE/RMSE log), and accuracy metrics $\delta_{1}$ (<1.25), $\delta_{2}$ (<$1.25^{2}$), and $\delta_{3}$ (<$1.25^{3}$).

### 4.2. Performance Evaluation

#### 4.2.1. Quantitative Comparison

This paper quantitatively evaluates the performance of the MsGf framework against several representative lightweight models on the KITTI and Make3D datasets, with comparative baselines including Monodepth2 [19] based on the ResNet50 architecture, the recently proposed Lite-Mono [30], and SwiftDepth [54].

As shown in Table 1 and Table 2, without employing any pre-training strategies, the MsGf framework significantly outperforms models such as Lite-Mono on geometric accuracy metrics, including Absolute Relative Error (AbsRel) and Root Mean Squared Error (RMSE). Most existing models are built upon standard residual modules, where traditional serial stacking or successive dilated convolutions tend to cause blurring and loss of detail. In contrast, the CDMs module in MsGf adopts parallel multi-scale convolutional kernel groups combined with atrous convolutions, enabling efficient multi-scale feature fusion while substantially reducing the number of parameters and enhancing feature extraction capability. This leads to notably better performance on geometric accuracy metrics (AbsRel, RMSE). Quantitatively, the CDMs module in MsGf reduces model parameters by 57% compared to Lite-Mono, while also decreasing floating-point operations (FLOPs), thereby improving inference speed accordingly. Furthermore, the SEGF module leverages the Sobel operator to extract directional edge features from RGB images and generates filtering kernels accordingly, which enhances structural consistency in the depth features and effectively suppresses artifacts. This results in a significant reduction in depth error metrics such as Squared Relative Error (SqRel) and RMSE log. The superior performance of MsGf on the accuracy threshold metrics $\delta_{1}$, $\delta_{2}$, and $\delta_{3}$ validates the synergistic effect of its multi-scale feature representation and edge-aware optimization. In comparison, the LGFI module in Lite-Mono, though designed for local–global feature interaction, lacks inherent edge awareness and artifact suppression capabilities—features that are fundamental to the SEGF module. Experimental results demonstrate that this lightweight design achieves an effective balance between parameter compression and feature preservation through synergistic optimization of multi-scale feature extraction and artifact suppression, thereby efficiently reducing model size.

#### 4.2.2. Visualization Comparison

**Figure 4:**
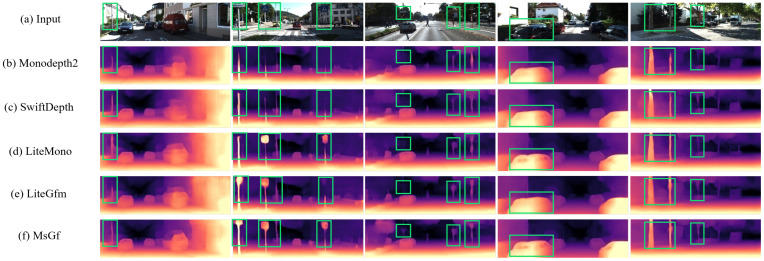
Visualize results on KITTI. Here are some depth maps generated by different lightweight models shown separately.

**Figure 5:**
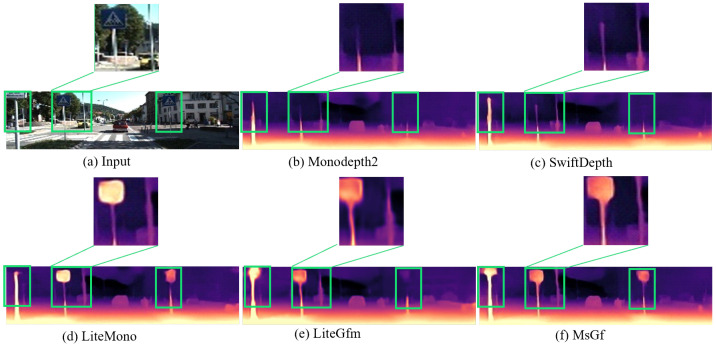
Visualize the results on the KITTI dataset. Here, the enlarged comparison of the middle sign and pole in the second column of Figure 4 is presented.

Furthermore, we conducted a visual comparison of depth maps generated by the MsGf framework against those produced by other representative models, including Monodepth2, Lite-Mono, and SwiftDepth, using samples from the Eigen split of the KITTI dataset, as shown in Figure 4 and Figure 5.

For vehicles with non-uniform color surfaces in columns 1 and 4, MsGf generates depth maps with sharper edges and more accurate depth transitions around the vehicles, as the guided filtering mechanism of the SEGF module effectively suppresses edge artifacts, achieving superior contour delineation compared to Lite-Mono and SwiftDepth. Regarding traffic signs in columns 2 and 3, MsGf captures finer details and maintains sharper edges; the synergistic effect of the CDMs and SEGF modules enables better preservation of structural information, yielding clearer and more accurate depth representations for such small objects. In areas with complex structures like walls and poles in columns 1, 2, and 5, MsGf exhibits fewer artifacts and more accurate depth predictions, where the lightweight multi-scale feature extraction of the CDMs module enhances complex scene handling while the SEGF module ensures robust edge and detail preservation. Under varying illumination conditions in columns 2, 4, and 5, MsGf demonstrates remarkable stability, producing consistent depth predictions. The boundary-aware loss function further enhances the retention of high-frequency structural details, resulting in more reliable depth maps for objects like poles, vehicles, and tree trunks across lighting variations. For uniform regions such as car glass surfaces in column 4, the CDMs module improves depth resolution. This enhancement, combined with the SEGF module’s edge reconstruction capability, produces more accurate and visually superior depth maps in textureless areas. Collectively, these visual comparisons demonstrate that MsGf outperforms other lightweight models, generating sharper and more accurate edges around vehicles, signs, and other objects with fewer artifacts in complex structures and uniform regions while maintaining stable performance under diverse illumination. The resulting depth maps are more complete and detailed, enhancing the structural integrity and realism of 3D scene reconstruction. These visual improvements corroborate quantitative results, validating the effectiveness of the proposed CDMs and SEGF modules in advancing monocular depth estimation.

### 4.3. Complexity and Speed Evaluation

This paper compares six mainstream lightweight models: Lite-Mono, SwiftDepth, R-MSFM, MViT-Depth [61], Monodepth2, and MsGf. By comparing parameters (Params), FLOPs, and inference speed, it evaluates MsGf’s complexity. As shown in Table 3, through the lightweight design of multiple convolution kernels and 1×1 operations in the CDMs module, as well as the guided filtering kernel generation mechanism in the SEGF module, the parameters of the MsGf framework are only 6.1% of those of Monodepth2 [19], significantly outperforming the existing lightweight models. At the same time, the inference speed of MsGf exceeds that of all the comparison methods. We have conducted additional experiments on real embedded hardware to validate our efficiency claims. Specifically, we evaluated our model on both Jetson Xavier and a high-performance GPU (Titan XP). Detailed results are now included in Table 4, Section 4.3 (Complexity and Speed Evaluation). The data demonstrate that the proposed lightweight design achieves a significantly higher frame rate on the Jetson platform compared to Monodepth2 and Lite-Mono, while maintaining performance advantages consistent with its low parameter count and computational complexity. This provides empirical validation for its lightweight and efficient design intended for practical deployment. Furthermore, additional experiments were conducted on real embedded hardware in this study. Specifically, our model was evaluated on both Jetson Xavier and a high-performance GPU (Titan XP). As demonstrated in Table 4, the proposed lightweight design achieves a substantially higher frame rate on the Jetson platform compared to Monodepth2 and Lite-Mono, while maintaining performance advantages consistent with its low parameter count and computational complexity.

### 4.4. Ablation Experiments

To verify the contribution of each module in the MsGf framework, we designed seven ablation experiments on the KITTI dataset (under the Eigen split standard) by removing or modifying certain network modules. The experiments quantified performance using seven metrics (Abs Rel, $\delta_{1}$, etc.) and used visualization to show each component’s impact on depth map quality. Figure 3 illustrates the flowchart of our key modules. Within the CDMs module, we designed and optimized a multi-scale feature extraction component, which effectively reduced the number of parameters and enhanced feature extraction capabilities. In the SEGF module, features were extracted using the Sobel operator, followed by two filtering operations to generate a filtering kernel, significantly reducing artifacts in depth maps. Prior to this, we experimented with alternative filtering kernel generation methods, which we refer to as the *new filtering kernels* and *old filtering kernels*. Furthermore, we employed a boundary-aware loss function, denoted as $L_{ba}$, to train our model, which further enhanced its performance. Details are as follows:

- Model 1, which only uses the basic network structure, without extra modules or mechanisms. It only includes a standard ResNet18 encoder and a simple decoder, without using any core modules. The encoder uses the standard ResNet-18 framework, and the decoder uses conventional deconvolution layers for upsampling.
- Model 2, which is the basic model with only the lightweight CDMs module.
- Model 3, which is the basic model with the CDMs module and the original self-attention mechanism, and is trained with the boundary-aware (BA) loss (see Section 3.4).
- Model 4, which is the basic model with the SEGF module to guide encoder features, and is trained with BA loss.
- Model 5, which uses the *old filtering kernels* to guide encoder features, and is trained with BA loss.
- Model 6, which is the basic model with CDMs and SEGF modules, but is not trained with BA loss.
- MsGf, which is the basic model with CDMs and SEGF, and is trained with BA loss.

The quantitative results of the ablation study are presented in Table 4. This paper evaluated seven distinct model configurations using seven metrics. Model 1, utilizing only the base network architecture without additional modules, exhibited the poorest performance. Performance improved significantly with the incremental addition of each component. The proposed MsGf model outperformed all ablation variants across every metric, demonstrating that each module within MsGf effectively enhances the network’s capability. For example, Model 2, which incorporates the CDMs module, reduced AbsRel from 0.125 to 0.124 and RMSE from 4.984 to 4.978 compared to Model 1. Model 4, which included the SEGF module and was trained with the boundary-aware loss, further decreased AbsRel to 0.123 and RMSE to 4.893. The final MsGf model achieved optimal performance with an AbsRel of 0.117 and an RMSE of 4.794, indicating a positive cumulative effect from all integrated components.

Notably, in Table 5, Model 1, which employs only the basic network structure, exhibits the highest parameter count. This is due to its encoder’s reliance on standard ResNet blocks with substantial parameters as the foundational building modules. In contrast, the proposed CDMs module incorporates a design that combines parallel multi-scale convolutions with efficient 1×1 convolutions, resulting in a parameter count significantly lower than that of the standard blocks. Therefore, when some of the standard ResNet blocks in the architecture are replaced with CDMs modules (as in Model 2), the total number of parameters does not increase; rather, it decreases markedly. This outcome convincingly demonstrates the effectiveness and advantage of the CDMs module in achieving model lightweighting.

**Figure 6:**
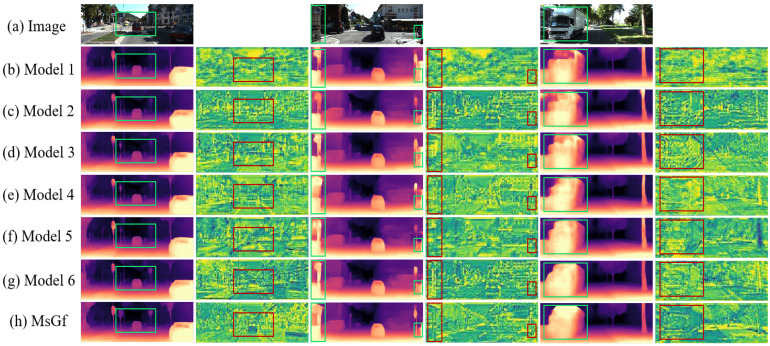
Comparison of depth maps and feature maps in different ablation experiments.

Furthermore, this paper clearly demonstrates the contribution of each module through visualization experiments. Figure 6 compares the depth maps and feature maps generated by different ablated models. When contrasting Model 1 with Model 2 and Model 3, the advantages of the lightweight CDMs module become evident. Due to its effective multi-scale feature extraction capability, Model 3, which incorporates the CDMs module plus an attention mechanism, produces significantly more accurate and detailed depth maps and feature maps, as seen in features like the house in the second image and the vehicle in the third image. The role of the SEGF module is demonstrated in Model 4, which generates sharper and more precise depth maps, exhibiting particularly outstanding performance in capturing complex details such as building contours. Model 5, employing an older filtering mechanism, shows some improvement in certain areas (e.g., the car and road sign in the first image, and the left house in the second image) but still suffers from detail loss and noise interference, such as artifacts between the right road sign and vehicles in the second depth map. In comparison, MsGf equipped with the SEGF module effectively reduces blurring and noise, thereby producing more accurate and visually superior depth maps. In summary, the ablation experiments confirm that each component of the MsGf framework contributes significantly to the overall performance. The CDMs module enhances multi-scale feature extraction, while the SEGF module effectively suppresses artifacts and preserves edge details. The combination of these modules, along with the boundary-aware loss function, collectively constitutes a lightweight yet powerful monocular depth estimation model.

### 4.5. Benefits of CDMs

To demonstrate the advantages of the lightweight CDMs module, we conducted ablation experiments. By comparing a series of indicators before and after the ablation, we verified the superiority of the CDMs module. In the lightweight cascaded structure, the CDMs module is stacked layer by layer, gradually integrating shallow and deep features. This enables the network to capture hierarchical features from different levels and enhance robustness in complex scenarios. The design of this module establishes a local–global feature collaborative perception mechanism. The lightweight structure ensures that the model can run efficiently on resource-constrained edge devices while maintaining accuracy.

As shown in Table 6, thanks to the depth separable convolution, multi-scale feature fusion, and channel compression of the CDMs module, the model parameters have decreased by 50%, FLOPs have reduced by 30%, and the single-frame inference speed has increased by 3.25 times. The multi-scale feature fusion strategy enhances the model’s feature extraction ability, and its lightweight design reduces parameters and computational costs. It improves the network’s ability to handle complex scenarios, thereby achieving better depth estimation performance.

### 4.6. Benefits of SEGF

**Figure 7:**
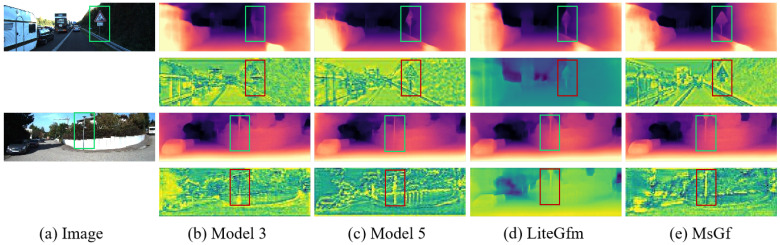
Comparison of depth maps and feature maps of Model 3, Model 5, LiteGfm, and MsGf frameworks.

Finally, to illustrate the superiority of the guided filtering module, we conducted an ablation study. Figure 7 presents a comparison of feature maps and depth maps with and without the SEGF module. Model 3, which lacks any filtering module, exhibits significant blurring and inaccuracies in its output, struggling to precisely render object details such as the road sign in the first image and the poles in the second image. Model 5 employs a variant filtering module, yet shows only limited improvement over Model 3; issues like detail loss and noise interference persist, particularly evident in the road sign and poles. Furthermore, we compared against LiteGfm, a lightweight model that also utilizes a filtering module. While its depth and feature maps demonstrate mitigated artifacts, the problem of detail loss remains inadequately resolved. In contrast, MsGf, equipped with the SEGF module, captures various image details more accurately. Features like road signs, vehicles, and building contours are rendered more clearly and precisely in its feature maps, effectively reducing blurring and noise. This comparison distinctly highlights the SEGF module’s pivotal role in enhancing the model’s ability to process image details and suppress artifacts, demonstrating its significant superiority.

## 5. Discussion

Our research findings demonstrate that the proposed MsGf framework is effective in reducing model parameters and eliminating artifacts.

By extensively employing 1×1 convolutions and discarding complex continuous dilated convolutions, the model parameter count was successfully reduced from 1.9 M to 0.8 M. However, the removal of continuous dilated convolutions weakened multi-scale feature extraction capabilities and impacted depth estimation accuracy. To address this, we introduce a parallel multi-scale convolution module, replacing the original 5×5 convolutions with 3×3 dilated convolutions. This maintains the ability to capture multi-scale information while further reducing parameters, effectively achieving model lightweighting as confirmed by experiments.

To tackle the artifact issue, we designed a guided image filtering module. This module utilizes the output of the network’s downsampling layer as the target image $I_{t}$ and the pooled image as the guidance image $I_{g}$. First, the Sobel operator is used to extract horizontal and vertical features from Ig. Subsequently, these features undergo a series of convolutional operations to generate horizontal and vertical feature maps. The kernel generated from the horizontal features is then applied to the vertical features in the first filtering step. Following this, a convolution operation is applied to the output of the first filtering step to generate a new kernel. Finally, this new kernel is used to filter the guidance image $I_{g}$ in a second filtering step, producing the final filtering kernel. For lightweighting purposes, 1×1 convolutions are predominantly used within this module. The two-step filtering operation progressively refines the process by leveraging the complementary information between depth features and RGB features, generating a filtering kernel adapted to the local image structure. This effectively suppresses artifacts while preserving edges and details, and compensates for the degradation in local feature extraction capability and accuracy resulting from the removal of continuous dilated convolutions and the parameter reduction.

**Figure 8:**
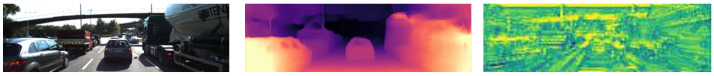
Examples of failure cases.

Based on the existing research, although the MsGf model was trained on the dynamic scenes of the KITTI dataset, it still has limitations. The failure cases shown in Figure 8 reveal a significant decline in the model’s performance when dealing with categories with a small sample size in the dataset, such as oil tankers. This situation not only limits the model’s generalization ability but also exposes potential challenges it may face in practical applications. Due to the limited number of samples of special vehicles like oil tankers in the KITTI dataset, the model has difficulty fully learning the features of these vehicles, resulting in large errors in actual detection. Moreover, the robustness of the MsGf model in handling different weather conditions and light changes also needs to be improved. For example, in strong light, backlight, or adverse weather conditions, the model’s detection accuracy may be affected because these conditions change the visible features of vehicles and the environment, thereby impacting the model’s recognition ability.

Future work will systematically focus on enhancing the generalization capability and robustness of lightweight depth estimation models in complex environments. First, we plan to construct a unified dataset integrating both indoor and outdoor scenarios. Building upon existing outdoor datasets, we will introduce a large-scale indoor dynamic environment dataset covering warehouses, underground parking lots, and office corridors, with varied lighting conditions and multi-floor configurations, to provide a more balanced and diverse data foundation for model training. Second, in terms of model architecture, we will explore a multi-task learning framework that jointly optimizes depth estimation, semantic segmentation, and camera pose estimation, thereby improving the model’s semantic awareness and geometric consistency in challenging environments such as repetitive textures, low-light conditions, and strong reflections. Finally, we will introduce meta-learning strategies to dynamically adjust loss weighting and feature distributions across different scenarios, facilitating zero-shot transfer of a single lightweight model to tasks such as autonomous driving. This approach will systematically address cross-domain challenges, including missed detection of oil tank trucks and false alarms caused by indoor highlights, ultimately enabling the development of an efficient, versatile, and highly adaptive depth estimation system.

## 6. Conclusions

This paper proposes a novel lightweight self-supervised monocular depth estimation framework. The aim of this project is to address the challenges of multi-scale feature extraction and artifact suppression in predicted depth maps. The CDMs module employs parallel multi-scale convolutional kernels to effectively capture both local and global features, thereby enhancing the network’s multi-scale feature extraction capability while maintaining a lightweight design. The SEGF module utilizes Sobel operators to generate horizontal and vertical gradient features, followed by a two-step filtering process to produce the filtering kernel. This approach effectively captures structural and edge features while reducing artifacts. Extensive experiments on the KITTI and Make3D datasets demonstrate that MsGf, with only 0.8 M parameters, outperforms current state-of-the-art methods. The MsGf framework not only delivers high-precision depth estimates but also exhibits excellent stability across diverse lighting conditions and complex scenes. It generates depth maps with sharper edges and fewer artifacts, making it highly suitable for real-time applications in resource-constrained environments such as autonomous driving and augmented reality.

The primary academic contribution of this study lies in the proposal of a lightweight architecture that integrates multi-scale convolution with edge-aware filtering, effectively addressing the challenges of structural preservation and artifact suppression in self-supervised depth estimation. This approach offers a novel solution for three-dimensional visual perception under resource-constrained conditions. Its compact and efficient design significantly reduces computational overhead while maintaining high accuracy, markedly improving the model’s applicability in real-world scenarios. In terms of social and industrial impact, the MsGf framework holds substantial potential for broad adoption and industrialization. It is suitable for application in domains highly sensitive to real-time performance, power consumption, and computational resources—such as autonomous driving, mobile augmented reality, and service robotics—by enhancing visual systems’ environmental understanding and decision-making capabilities in complex settings while reducing hardware deployment costs.

In future work, we will further optimize the model architecture and explore its integration with multi-task learning and cross-modal perception systems to enhance robustness and adaptability in broader scenarios. These efforts aim to provide foundational support for achieving efficient and generalizable machine perception. 

## Figures and Tables

**Table 1:** The quantitative results of MsGf with some recent representative methods on the eigen split of the KITTI dataset. The best results are presented in bold for each category, with the second-best results underlined. The upward arrow (↑) indicates that higher values are better, while the downward arrow (↓) indicates that lower values are better.

Methods	AbsRel↓	SqRel↓	RMSE↓	RMSE log↓	δ1↑	δ2↑	δ3↑	Param
Zhou [55]	0.183	1.595	6.709	0.270	0.734	0.902	0.959	31.6 M
GeoNet [56]	0.155	1.296	5.857	0.233	0.793	0.931	0.973	31.6 M
DDVO [57]	0.151	1.257	5.583	0.228	0.810	0.936	0.974	28.1 M
EPC++ [58]	0.141	1.029	5.350	0.216	0.816	0.941	0.976	33.2 M
MonoDepth2-ResNet18 [19]	0.132	1.044	5.142	0.210	0.845	0.948	0.977	14.3 M
MonoDepth2-ResNet50 [19]	0.131	1.023	5.064	0.206	0.849	0.951	0.979	32.5 M
R-MSFM3 [59]	0.128	0.965	5.019	0.207	0.853	0.951	0.977	3.5 M
R-MSFM6 [59]	0.126	0.944	4.981	0.204	0.857	0.952	0.978	3.8 M
Lite-Mono-tiny [30]	0.125	0.935	4.986	0.204	0.853	0.950	0.978	2.1 M
Lite-Mono-small [30]	0.123	0.919	4.926	0.202	0.859	0.951	0.977	2.5 M
Lite-Mono [30]	0.121	0.876	4.918	0.199	0.859	0.953	0.980	3.1 M
SwiftDepth-small [54]	0.132	1.040	5.148	0.210	0.846	0.948	0.976	3.6 M
SwiftDepth [54]	0.128	1.020	5.093	0.205	0.850	0.951	0.978	6.4 M
LiteGfm-small [60]	0.123	0.924	4.992	0.199	0.858	0.953	0.980	1.7 M
LiteGfm [60]	**0.117**	0.870	4.797	**0.194**	0.870	0.957	**0.981**	1.9 M
MViT-Depth-tiny [61]	0.131	0.947	5.114	0.207	0.853	0.950	0.977	1.8 M
MViT-Depth-small [61]	0.128	0.926	4.938	0.203	0.857	0.951	0.978	2.8 M
MViT-Depth [61]	0.122	0.887	4.885	0.197	0.862	0.953	0.980	6.3 M
**MsGf (ours)**	**0.117**	**0.871**	**4.794**	**0.194**	**0.871**	**0.958**	**0.981**	**0.8 M**

**Table 2:** The depth estimation results on the Make3D and NYU Depth V2 datasets. The best scores are in bold. The upward arrow (↑) indicates that higher values are better, while the downward arrow (↓) indicates that lower values are better.

Methods	AbsRel↓	SqRel↓	RMSE↓	RMSE log↓
MonoDepth2	0.364	4.468	7.976	0.179
Lite-Mono	0.352	4.085	7.756	0.174
Swiftdepth	0.359	3.842	7.710	0.180
**MsGf (ours)**	**0.335**	**3.651**	**7.488**	**0.167**
**Methods**	**AbsRel↓**	**RMSE↓**	***δ*1↑**	***δ*2↑**
MonoDepth2	0.739	**1.338**	0.440	0.727
Lite-Mono	0.486	2.019	0.403	0.686
**MsGf (ours)**	**0.387**	1.355	**0.442**	**0.735**

**Table 3:** Model complexity and speed evaluation. PARAMS denotes the number of parameters. FLOPS are floating points of operations. Speed is inference time. The best results are presented in bold for each category.

Methods	Encoder			Decoder			Full Model		
	Params (M)	FLOPS (G)	Speed (MS)	Params (M)	FLOPS (G)	Speed (MS)	Params (M)	FLOPS (G)	Speed (MS)
MonoDepth2	11.2	4.5	0.8	3.1	3.5	0.9	14.3	8.0	1.7
R-MSFM3	0.7	2.4	0.2	2.8	14.1	3.4	3.5	16.5	3.6
R-MSFM6	0.7	2.4	0.2	3.1	28.8	5.6	3.8	31.2	5.8
MViT-Depth-tiny	1.0	0.7	1.5	0.8	0.8	0.3	1.8	1.5	1.8
MViT-Depth-small	1.9	1.8	1.9	0.9	1.0	0.4	2.8	2.8	2.3
MViT-Depth	5.0	3.6	2.2	1.3	1.1	0.4	6.3	4.7	2.6
Lite-Mono-tiny	2.0	2.4	1.6	0.2	0.5	0.2	2.2	2.9	1.8
Lite-Mono-small	2.3	4.1	2.0	0.2	0.7	0.2	2.5	4.8	2.2
Lite-Mono	2.9	4.4	2.1	0.2	0.7	0.2	3.1	5.1	2.3
SwiftDepth-small	3.0	1.5	1.7	0.6	2.1	0.2	3.6	3.6	1.9
SwiftDepth	5.6	2.4	2.2	0.8	2.5	0.2	6.4	4.9	2.4
LiteGfm-small	1.5	1.9	1.2	0.2	0.5	0.9	1.7	2.4	2.1
LiteGfm	1.7	3.3	1.7	0.2	0.7	0.9	1.9	4.0	2.6
**MsGf (ours)**	**0.6**	**2.2**	**0.1**	**0.2**	**0.7**	**0.7**	**0.8**	**2.9**	**0.8**

**Table 4:** Comparison of inference speed on Titan XP and Jetson AGX Xavier. The best results are presented in bold for each category.

Methods	Encoder		Decoder		Full Model		Speed (ms)	
	Params (M)	FLOPS (G)	Params (M)	FLOPS (G)	Params (M)	FLOPS (G)	Titan XP	Jetson Xavier
Monodepth2	11.2	4.5	3.1	3.5	14.3	8.0	3.8	14.3
R-MSFM3	0.7	2.4	2.8	14.1	3.5	16.5	7.8	22.3
R-MSFM6	0.7	2.4	3.1	28.8	3.8	31.2	13.1	41.7
MonoViT-tiny	5.6	7.8	4.7	15.9	10.3	23.7	13.5	47.4
Lite-Mono-tiny	2.0	2.4	0.2	0.5	2.2	2.9	3.3	12.7
Lite-Mono-small	2.3	4.1	0.2	0.7	2.5	4.8	4.3	19.2
Lite-Mono	2.9	4.4	0.2	0.7	3.1	5.1	4.5	20.0
**MsGf (ours)**	0.6	2.2	0.2	0.7	**0.8**	**2.9**	**1.5**	**11.6**

**Table 5:** Results of ablation experiments in the seven models. ✔ indicates the use of this module, while \ indicates that the module is not used. The upward arrow (↑) indicates that higher values are better, while the downward arrow (↓) indicates that lower values are better.

Model	CDMs	SEGF	BA Loss	Params (M)↓	AbsRel↓	SgRel↓	RMSE↓	RMSE log↓	$\delta_{1}$↑	$\delta_{2}$↑	$\delta_{3}$↑
Model 1	\	\	\	1.936	0.125	0.978	4.984	0.202	0.859	0.952	0.978
Model 2	✔	\	\	0.866	0.124	0.946	4.978	0.200	0.863	0.954	0.979
Model 3	✔	\	✔	0.996	0.123	0.943	4.908	0.199	0.866	0.955	0.980
Model 4	\	✔	✔	1.638	0.123	0.937	4.893	0.198	0.866	0.956	0.980
Model 5	✔	*old*	✔	0.832	0.121	0.935	4.875	0.198	0.867	0.956	0.980
Model 6	✔	✔	\	0.828	0.120	0.924	4.856	0.196	0.868	0.957	0.981
**MsGf**	✔	✔	✔	**0.828**	**0.117**	**0.881**	**4.794**	**0.194**	**0.871**	**0.958**	**0.981**

**Table 6:** Complexity-speed performance analysis of Model 4 and the MsGf framework. The downward arrow (↓) indicates that lower values are better.

Model	Params (M)↓	FLOPs (G)↓	Speed (ms)
Model 2	1.0	1.7	1.5
Model 3	0.9	2.9	1.2
Model 4	1.6	4.0	2.6
MsGf	0.8	2.8	0.8

## Data Availability

The data that support the findings of this study are available from the corresponding author upon reasonable request.
